# Proposed Safety Guidelines for Patient Assistants in an Open MRI Environment

**DOI:** 10.3390/ijerph192215185

**Published:** 2022-11-17

**Authors:** Sukhoon Oh, Seon-Eui Hong, Hyung-Do Choi

**Affiliations:** 1 Bio-Chemical Analysis Team, Korea Basic Science Institute, Cheongju 28119, Republic of Korea; 2 Radio & Satellite Research Division, Electronics and Telecommunications Research Institute (ETRI), Daejeon 34129, Republic of Korea

**Keywords:** open MRI system, patient assistant, planar RF coil, SAR, safety guideline

## Abstract

The wide-open side of an open magnetic resonance imaging (MRI) system allows a patient to easily contact the patient assistant during MRI scans. A wide-open-shaped magnet is highly effective when interventional procedures are necessary. Patient assistants can provide comfort by holding a part of the patient’s body. Because current regulations or guidelines are concerned with only patient radio frequency (RF) safety, investigations on the safety of patient assistants exposed to high-magnetic field MRI (up to 1.2 T) are required. In this study, five different poses of patient assistants were numerically simulated at a 1.2 T open MRI system to determine the impact of poses on the RF exposure level. The 10-g averaged specific absorption rate (SAR) levels were analyzed for the poses of each patient assistant wearing gloves. Compared with the patient, up to 29.8% of the patient SAR was observed in the patient assistant. When the patient assistant wore latex gloves, a 63.7% reduction in the 10-g averaged SAR level was observed, which could be a remedy to minimize possible RF hazards. To prevent possible RF hazards during MRI scans, certain clauses regarding the patient assistant’s poses or wearing gloves must be added to the existing MRI screening forms.

## 1. Introduction

Magnetic resonance imaging (MRI) is a non-invasive imaging modality that produces detailed three-dimensional anatomical images. The MRI system can be utilized for neurologic, cardiac, and musculoskeletal disorders because of its excellent contrast of soft tissues and non-invasive nature. Therefore, the use of MRI systems for diagnosis and research purposes has increased considerably.

The MRI system can be categorized into two types of magnets that are closed or open magnet types. The magnetic field strength (B_0_) of a closed MRI system is generally higher than that of the open-magnet type, which is higher than 1.5 T. The most significant advantage of the closed MRI system is its superior signal-to-noise ratio (SNR) because of the higher B_0_. However, the closed space may cause claustrophobia in patients that may result in a psychologically disturbed condition, particularly in younger people. Eventually, the total MRI scan time may increase because of patient movement during MRI scans. However, fewer chances of claustrophobia are expected in an open MRI system mainly because of the open shape of the magnet. The magnets of the open MRI system are located at the chest and back of the patient, such that the patient feels comfortable relative to the closed magnet. Because the sides of the open MRI system are widely open, it is possible to access the patient even during MRI scans. Accessing a patient in an open MRI system is occasionally necessary for intervention procedures. The support provided by patient assistants during MRI scans can afford considerable psychological comfort to patients, enabling the timely completion of all the required MRI scans. This is an important advantage of an open MRI system, even though its SNR is lower than that of a closed magnet.

The excitation magnetic field (B_1_^+^) must be formed by the transmission of radio frequency (RF) energy to a human body for MRI signal generation. However, the B_1_^+^ field also affects the RF energy deposition during the MRI scans because the induced electric field directly causes the specific absorption rate (SAR). If the SAR associated with temperature rise is underestimated during MRI scans, it may cause thermal hazards owing to RF exposure. A patient, worker, or even a patient assistant could be affected by thermal hazards. Safety limits for RF exposure of the human body to time-varying electromagnetic fields (EMFs) are provided by the International Commission on Non-Ionizing Radiation Protection (ICNIRP) [1,2,3]. In particular, for RF exposure of a patient during a clinical MRI scan, ICNIRP published a statement in 2004 [4] and an update in 2009 [5]. The international standard IEC 60601–2–33 [6] established basic safety and essential performance requirements for MRI systems to provide protection to patients. The International Society for Magnetic Resonance in Medicine (ISMRM) has recently endorsed the best practice or guidance on RF safety-related MRI experiments and/or numerical assessments [7,8]. Several studies have assessed RF safety during MRI scans for workers [9,10,11,12] and patients, including adults with implanted medical devices [13,14,15,16,17] and the general public [18,19,20,21].

Most regulations or RF safety guidelines for MRI concern only the RF exposure of a patient, with or without implant devices or workers [6,7,8]. However, there have been few studies and guidelines on RF exposure of patient assistants during MRI scans. Access of patient assistants to patients is enabled by the shape of the open MRI system, even during MRI scans. Therefore, it is necessary to evaluate the RF energy exposure of patient assistants. A previous study [22] has shown that several variables can affect the distribution of SAR in a patient assistant, including distance from the head or body transmit RF coil and configuration of interventional treatments at 0.3 T in an open MRI system. In this study, we numerically investigated RF energy exposures of patient assistants using planar-shaped RF coil models, which are widely used to transmit RF coil type, particularly at 1.2 T in an open MRI system under various exposure conditions. The SAR levels tend to increase in proportion to the square of the increase in B_0_; therefore, it is necessary to examine the SAR levels of a patient assistant in a 1.2 T MRI environment because the strongest B_0_ strength in an open MRI system is 1.2 T. Based on our investigations, we propose guidelines to protect the patient assistants from the possible RF hazards during MRI scans.

## 2. Materials and Methods

**Figure 1:**
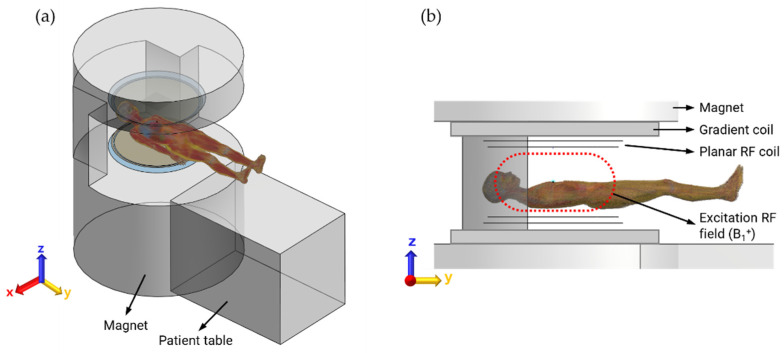
(**a**) View of an open MRI used in this study; (**b**) Top and bottom symmetrically placed magnet, gradient coil, and RF coil in z-direction. Dashed red line indicates the area of transmit RF field (B_1_^+^) that is induced by RF coils.

An open MRI system including a magnet, gradient coil, and planar-type RF coil was numerically modeled, as shown in Figure 1, to mimic the basic environment of RF exposure of the patient assistant during MRI scans. We assigned the material properties of the magnet and gradient coil to air (electrical conductivity *σ*, 0 S/m, and relative permittivity *ε_r_*, 1), implying that the magnet and gradient coil do not affect the overall results. However, the space for various poses of the patient assistant needs to be practically secured by placing the magnet and gradient coil. As shown in Figure 1, both sides of the magnet structure enable a patient assistant to approach the patient, even during MRI scans. The detailed setting of the transmit RF coil for RF exposure analysis is described in the following section.

### 2.1. Transmit RF Coil Modeling

**Figure 2:**
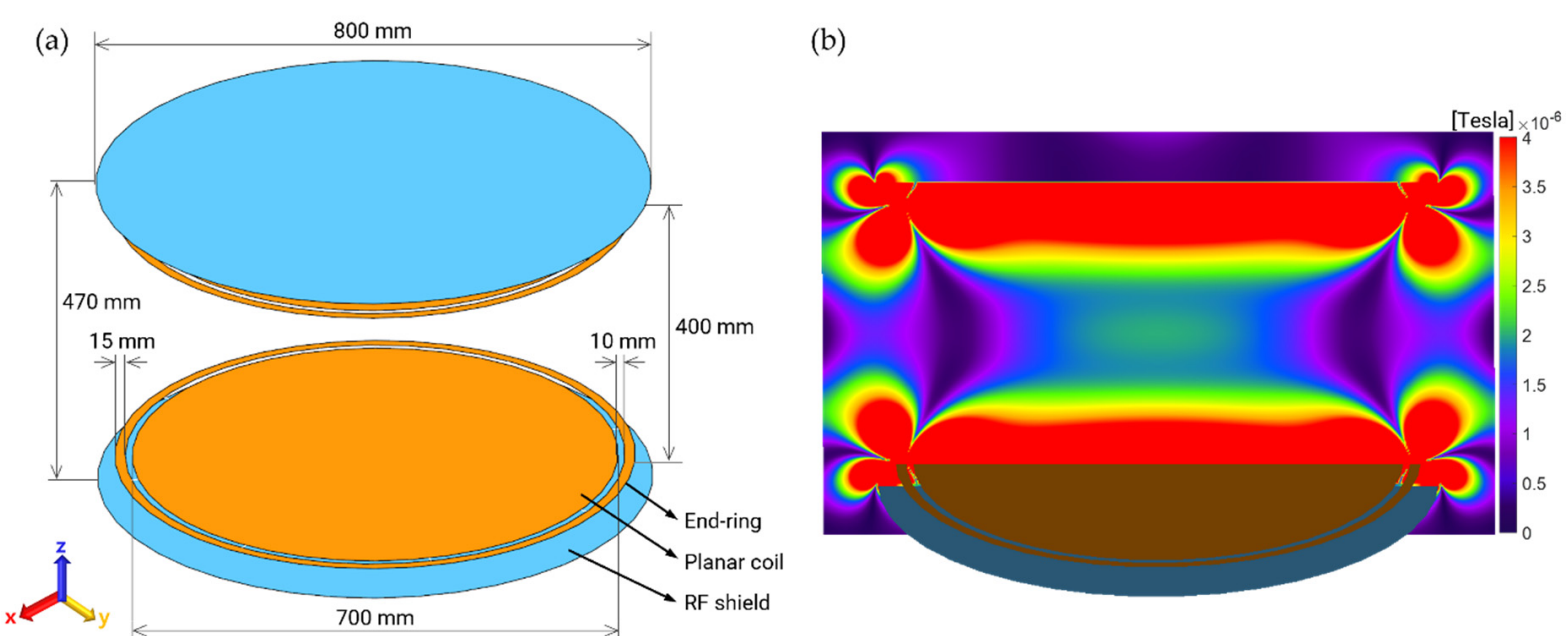
(**a**) Numerical modeling of the planar T_X_ RF coil. The electromagnetic field generating the MR imaging signal forms in between two plana coil elements; (**b**) Excitation magnetic field (B_1_^+^). The applied RF power was scaled to create 2 μT of B_1_^+^ at the center of magnet (upper elements of Tx RF coil were removed for better visualization).

The only role of the transmit (Tx) RF coil in the MRI system is the generation of an imaging signal in the patient’s body based on application of an appropriate amount of RF power through the Tx RF coil [23]. Creating a uniform excitation magnetic field (B_1_^+^, as shown by the dashed red line in Figure 1) over a larger area of the human body is a fundamental requirement for a Tx RF coil. In addition, the quadrature driving of the RF coil can lower the total amount of RF power, thus creating a circular polarized (CP mode) B_1_^+^ field, resulting in a higher signal-to-noise ratio (SNR) than that in a linear mode. In this study, we introduced one of the planar-shaped Tx RF coils from previous studies that showed a significantly larger B_1_^+^ field in the CP mode [24,25], as shown in Figure 2a. The planar Tx coil is top–down symmetrically (in the z-direction) and composed of RF shielding plates (800 mm in diameter), end-ring (15 mm in width), and planar RF coils (with 10 mm gap between end-ring and coil, with 400 mm space between the coils). Eight constant current sources were circularly placed at the gap in equispaced order (i.e., at every 45° of phase) to create a B_1_^+^ field around the *z*-axis. There was a 90° phase difference between the top and bottom of the RF coil element that resulted in a CP mode in the human body. The planar Tx RF coil was driven at 51 MHz, the resonance frequency of 1.2 T MRI system, such that the human body within the B_1_^+^ field and electric field is exposed. A patient assistant must also be exposed to electromagnetic (EM) fields because the patient assistant is located sufficiently close to the EM fields. As shown in Figure 2b, we confirmed suitable functioning of the planar Tx coil by observing the excited magnetic field (B_1_^+^) at 51 MHz under a cylindrical phantom loading condition. The diameter and depth of the cylindrical phantom were 720 mm and 360 mm, respectively. The electrical conductivity, relative permittivity, and material density were also assigned to mimic the muscle tissue at 51 MHz (0.68 S/m, 76.6, and 1090.4 kg/m^3^, respectively). Note that the cylindrical phantom was removed, as shown in Figure 2a, for better visualization.

The amount of applied RF power of the Tx RF coil was scaled to create the same magnitude of B_1_^+^, for example, 2 μT, at the isocenter of the magnet (Figure 2b) for each pose of the patient assistant. A few calibration steps are generally performed prior to real MRI scans. One of the important calibrations is the adjustment of the RF power to achieve a specific flip angle (i.e., 90°) of magnetic spins in the human body [26]. The amount of RF power must differ from the loading conditions of the Tx RF coil, including the patient mass and the surrounding environment of the patient. The RF exposure level for each case can be quantitatively and reasonably compared by maintaining the same magnitude as B_1_^+^.

### 2.2. Numerical Human Body Models and RF Exposure Scenarios

Two numerical human body models were employed to assess RF exposure. First, an 11–year–old female model (Billie), a child patient, was placed within the planar RF coil in a lying down position, as shown in Figure 1a. The Billie model is composed of 75 different biological tissues with appropriate tissue properties, such as electrical conductivity (S/m), relative permittivity, and material density (kg/m^3^) at a frequency of 51 MHz. The Posable Duke model was employed as the patient assistant. Duke is a 34-year-old adult male patient assistant with 77 biological tissues, as shown in Table 1. Suitable tissue properties were assigned to the Duke model at 51 MHz. Additionally, the changes in the poses of a numerical body model are available at Duke. Numerical human body models, including these two, are available from the Foundation for Research on Information Technologies in Society (IT’IS) [27,28].

**Figure 3:**
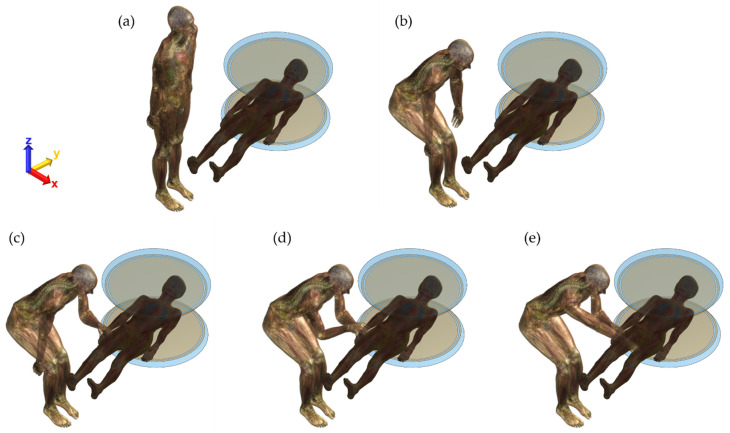
Various poses of patient assistant and the corresponding RF exposure; (**a**) Pose 1: standing; (**b**) Pose 2: sitting next to a patient; (**c**) Pose 3: touching a patient’s hand with one hand of patient assistant; (**d**) Pose 4: two hands stacked up; and finally, (**e**) Pose 5: patient’s hand and thigh touched with two hands.

Five different scenarios of patient assistants were defined by changing the poses and patient contact conditions using the posable Duke model to examine the RF exposure, as shown in Figure 3, because these poses were performed occasionally, especially in the open MRI system. In cases of standing (Figure 3a) and sitting (Figure 3b) next to a patient, a low level of RF exposure was expected. The presence of a patient assistant reassures a patient, enabling open MRI scans. In other cases, the patient assistant also touched the patient with one hand (Figure 3c), had two hands stacked up (Figure 3d), and finally, had two hands on the patient’s hand and thigh (Figure 3e).

EM field simulations of RF exposure of patient assistants were performed using Sim4Life software (Zurich Med Tech AG, Switzerland), a finite-difference time-domain (FDTD)-based EM field solver. All computations were conducted on a high-performance computing (HPC) system that was equipped with a GPU (two NVIDIA Quadro RTX A6000s), CPU (Intel Xeon 6126), and 704 GB of system memory. The grid size of each pose was 4 × 4 × 4 for all the EM field simulations. The total number of cells in each case was approximately 290 mega cells, such that the required computation time was approximately 1 h for each case using the above HPC system.

The 10-g averaged SAR [6] was separately collected from the patient assistant and the patient as the RF exposure level indicator for each pose.

## 3. Results and Discussion

The maximum 10-g averaged SAR values from the patient assistant and patient for the five different poses are summarized in Table 2. As previously mentioned, the RF power applied to the planar Tx RF coil was scaled individually, such that the magnitude of B_1_^+^ at the center of the magnet of each pose was maintained at 2 μT. The lowest maximum 10-g averaged SAR level was found for Pose 2 (sitting next to the patient, Figure 3b), which is 0.1041 W/kg. The highest maximum 10-g SAR level was measured for Pose 3 (wherein one hand of the patient’s assistant touched the patient, as shown in Figure 3c), which was 5.2606 W/kg. This is approximately 50.5 times higher than the lowest maximum 10-g SAR level of the patient assistant.

The other two poses (4 and 5) of the patient assistant showed an approximately 11% lower 10-g SAR level than Pose 3. This is because of the total mass difference within the exposed electric field of the planar Tx RF coil, particularly around the edge of the coil. A smaller mass tends to result in greater RF exposure. In addition, the induced SAR level of the patient assistant would worsen if a patient assistant was electrically connected to the patient (i.e., touching each other). Based on this interpretation, the total mass of touching with one hand (Pose 3) results in a higher level of RF exposure than touching with two hands (Poses 4 and 5), as summarized in Table 2. Furthermore, no significant differences in the maximum 10-g SAR levels in patients were found, regardless of the pose of the patient assistant.

**Figure 4:**
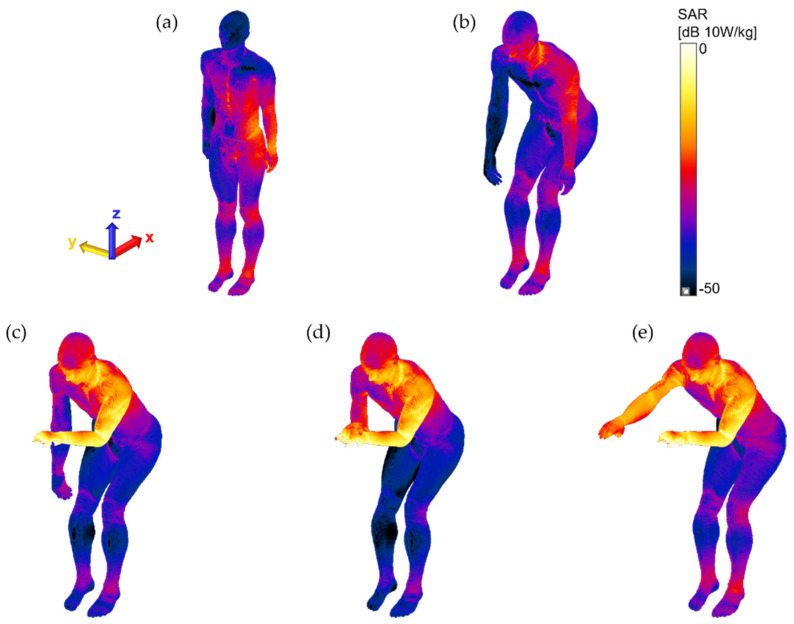
Surface SAR of each pose of patient assistant for visual observation of the distribution of SAR for each pose in decibel scale (relative scale to 10 W/kg).

**Figure 5:**
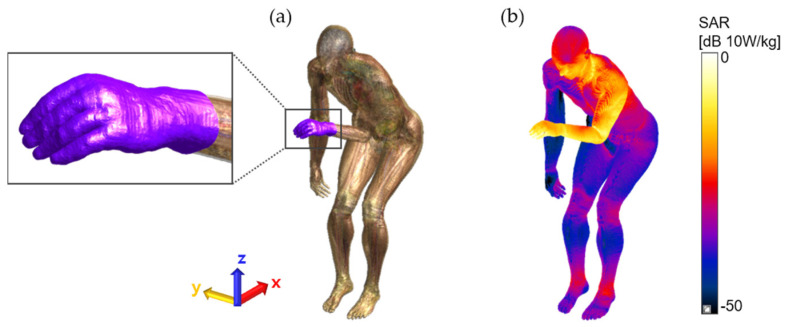
(**a**) Wearing latex glove to reduce the 10-g averaged SAR. (**b**) 63.7% of 10-g averaged SAR, in comparison to the maximum 10-g averaged SAR, was observed (see Table 2, Pose 3 of patient assistant).

The surface SAR of the patient assistant was also examined to visually observe the distribution of the SAR for each pose in decibels (relative scale to 10 W/kg), as shown in Figure 4. The locations of the patient assistants were fixed near the edge of the planar Tx RF coil, but the poses were adjusted according to the scenario. This affects the distribution of the surface SAR, such that a slightly higher maximum 10-g SAR level was measured in the standing position than in the sitting position in the case of non-touching poses. An extremely strong EM field was formed at the edge of the coil (Figure 2b). For all touching poses (Pose 3–5), the maximum 10-g SARs were found at the fingertip of the left hand in case of an electrical connection to the patient. This signifies that touching the patient’s body with a bare hand by a patient assistant during MRI scans may lead to a higher level of RF exposure in the patient assistant. For this reason, we suggest wearing electrically insulated gloves, such as latex or cotton gloves, preventing direct contact with the patient’s body when the patient assistant needs to be involved in MRI scans. We performed an additional EM field simulation to determine whether wearing gloves reduced RF exposure. Because the highest maximum 10-g SAR was found for Pose 3 (touching the patient’s body with one hand, Figure 3c and Figure 4c), the patient assistant’s left hand was slightly lifted up to avoid touching the patient’s body. No other body part of the patient assistant was adjusted while the 3-mm-thick latex glove layer was placed between them. The material properties of latex glove were 0 S/m electrical conductivity and 1.6 relative permittivity, as shown in Figure 5a in purple color. As shown in Figure 5b, a slight alteration of the surface SAR was observed, compared with Figure 3c, at the thighs, knees, ankles, and specifically at the tip of the left hand in case of wearing the glove. The glove-wearing case showed a 63.7% lower maximum 10-g SAR (1.9098 W/kg) than the case without wearing gloves (Pose 3, 5.2606 W/kg).

While assisting patients by holding their body parts during MRI scans, the patient assistants should be aware of the possible risk of RF exposure. In addition, insulating gloves must be worn to minimize the possibility of unwanted adverse events, as confirmed by a 63.7% reduction in the SAR level.

## 4. Conclusions

In this study, we investigated the amount of RF exposure in five different poses of a patient assistant during MRI scans at 1.2 T in an open MRI system to determine the impact of poses on the level of RF exposure. The planar Tx RF coil near the patient assistant induces a higher 10-g averaged SAR in the patient assistant, which is approximately 50 times higher when the patient assistant holds the patient’s body compared to the sitting pose. We have found that it can be reduced by approximately 63.7% if the patient assistant wears electrically insulating gloves as a remedy to possible RF hazards.

The screening forms of patients can be found in any MRI scanning room. Based on our study, we suggest adding certain clauses to the existing MRI screening forms as follows:

- The patient assistants must not hold the patient’s body during the MRI scans unless otherwise instructed;
- If patient contact is required, the patient assistants must wear electrically insulated gloves whenever they are in the MRI scan room;
- The patient assistants must not touch any cables, equipment, or electronics of the MR scanner, even wearing insulating gloves.

## Figures and Tables

**Table 1:** Numerical human body models.

Role	Name (Gender)	Age	Height (cm)/Weight (kg)	# of Tissue
Patient assistant	Duke (male)	34	173/70	77
Patient	Billie (female)	11	146/36	75

**Table 2:** Maximum 10-g averaged SAR values for five different poses of patient assistant and their locations.

Subject	Pose ^1^	Max. 10 g SAR [W/kg] ^2^	Location of Max. 10 g SAR ^2^
Patient assistant	1: standing	0.1881	left pelvis
	2: sitting	0.1041	left shoulder–neck
	3: one hand placed	5.2606	left hand (at the edge of RF coil)
	4: two hands stacked up	4.7715	left hand (at the edge of RF coil)
	5: two hands on hand and thigh	4.5892	left hand (at the edge of RF coil)
Patient	1: standing	18.0826	left thumb–thigh (at the edge of RF coil)
	2: sitting	18.2466	left thumb–thigh (at the edge of RF coil)
	3: one hand placed	18.6861	left thumb–thigh (at the edge of RF coil)
	4: two hands stacked up	18.6014	left thumb–thigh (at the edge of RF coil)
	5: two hands on hand and thigh	17.3428	left thumb–thigh (at the edge of RF coil)

^1^ Poses of patient assistant; ^2^ Max. 10-g averaged SAR values and approximate locations of each subject and pose.

## Data Availability

Not applicable.
